# Epigenetic Effects of Resveratrol on Oncogenic Signaling in Breast Cancer

**DOI:** 10.3390/nu16050699

**Published:** 2024-02-29

**Authors:** Lucinda Kurzava Kendall, Yuexi Ma, Tony Yang, Katarzyna Lubecka, Barbara Stefanska

**Affiliations:** 1 Department of Nutritional Sciences, Purdue University, West Lafayette, IN 47907, USA; 2 Food, Nutrition and Health Program, Faculty of Land and Food Systems, The University of British Columbia, Vancouver, BC V6T 1Z4, Canada; 3 Department of Biomedical Chemistry, Medical University of Lodz, 92-215 Lodz, Poland

**Keywords:** resveratrol, DNA methylation, Wnt, Hedgehog, oncogenes, chromatin, cancer

## Abstract

The crosstalk between oncogenic signaling pathways plays a crucial role in driving cancer development. We previously demonstrated that dietary polyphenols, specifically resveratrol (RSV) and other stilbenoids, epigenetically target oncogenes for silencing via DNA hypermethylation in breast cancer. In the present study, we identify signal transduction regulators among RSV-hypermethylated targets and investigate the functional role of RSV-mediated DNA hypermethylation in the regulation of Hedgehog and Wnt signaling. Non-invasive ER-positive MCF-7 and highly invasive triple-negative MCF10CA1a human breast cancer cell lines were used as experimental models. Upon 9-day exposure to 15 µM RSV, pyrosequencing and qRT-PCR were performed to assess DNA methylation and expression of *GLI2* and *WNT4*, which are upstream regulators of the Hedgehog and Wnt pathways, respectively. Our results showed that RSV led to a DNA methylation increase within *GLI2* and *WNT4* enhancers, which was accompanied by decreases in gene expression. Consistently, we observed the downregulation of genes downstream of the Hedgehog and Wnt signaling, including common targets shared by both pathways, *CCND1* and *CYR61*. Further analysis using chromatin immunoprecipitation identified increased H3K27 trimethylation and decreased H3K9 and H3K27 acetylation, along with abolishing OCT1 transcription factor binding. Those changes indicate a transcriptionally silent chromatin state at *GLI2* and *WNT4* enhancers. The inhibition of the Wnt signal transduction was confirmed using a phospho-antibody array that demonstrated suppression of positive and stimulation of negative Wnt regulators. In conclusion, our results provide scientific evidence for dietary polyphenols as epigenetics-modulating agents that act to re-methylate and silence oncogenes, reducing the oncogenic signal transduction. Targeting such an action could be an effective strategy in breast cancer prevention and/or adjuvant therapy.

## 1. Introduction

Breast cancer remains the leading cancer and the second cause of cancer-related deaths in women in North America despite tremendous progress in screening, early detection, and treatments [1]. Common features of breast cancer are aberrant patterns of the epigenome with changes in DNA methylation, as the most abundantly studied [2,3] and connected even compared to the socio-economic aspects [4].

Attention in the field of cancer epigenetic research has been largely focused on the increase in DNA methylation of tumor suppressor genes as an early event in cancer development [5]. It is well accepted that promoter hypermethylation leads to the silencing of tumor suppressor genes, such as, for example, *PTEN*, *APC*, and *RARbeta2* in breast cancer, and that the reversal of the repression of these genes results in anti-cancer effects [6]. Hence, the main focus in epigenetic pharmacology was on developing strategies that will inhibit DNA methylation and remove aberrant methylation marks from tumor suppressor genes to produce anti-cancer effects. However, technological advances allowed for genome-wide studies of DNA methylation patterns in cancer, which showed that some hypermethylated promoters are actually associated with active transcription rather than silencing [7]. In pancreatic cancer, this phenomenon was mechanistically explained by the preferential binding of the NFATc1 transcription factor to hypermethylated DNA, followed by the activation of the transcriptional machinery [7]. Recent decades of genome-wide studies of the DNA methylation landscape in cancer also show that DNA hypomethylation of promoters and other gene regulatory regions, like enhancers, is as prevalent in cancer as hypermethylation [8,9,10]. Interestingly, the hypomethylated genes share common functions relevant to cancer including cell proliferation, migration, and invasion. We previously reported that genes differentially methylated between non-invasive LNCaP and highly invasive PC-3 prostate cancer cell lines include, predominantly, a large number of genes that are hypomethylated in invasive cells and may account for highly invasive potential [11]. Our studies on clinical samples from liver cancer provided further evidence of organized hypomethylation events within regulatory regions of genes involved in cellular transformation [8]. We provided strong mechanistic support for the oncogenic functions of some of the hypomethylated genes, including *RASAL2* and *NENF* [9]. Loss of DNA methylation marks at candidate genes was also shown to be critical in invasiveness and metastasis in breast and prostate cancer in earlier studies [12,13]. These pieces of evidence indicate the importance of developing therapeutic strategies that target the reversal of demethylation of oncogenes and pro-metastatic genes by leading to their re-methylation and silencing.

Interestingly, naturally derived compounds have been shown by us and others to modify the epigenome and bring about profound phenotypic changes at non-toxic doses [14,15,16]. Our previous findings demonstrated that polyphenols from the stilbenoid class, including resveratrol (RSV), lead to increased levels of DNA methylation at regulatory regions of hundreds of oncogenes in breast cancer, thereby decreasing their transcriptional activities [16]. We identified numerous genes linked to key oncogenic pathways among polyphenol hypermethylated targets, including *MAML2*, an activator of the NOTCH oncogenic signaling pathway [16]. Both RSV and its analog, pterostilbene (PTS), induced hypermethylation and the silencing of *MAML2*. This led to the inhibition of downstream components of the NOTCH signaling pathway, indicating pathway suppression [16]. Knockdown of *MAML2* mimicked RSV and PTS anti-cancer action, implying an important functional role of the epigenetic changes within the NOTCH pathway [16]. A similar pattern of changes in DNA methylation landscapes, including an increase in DNA methylation of oncogenes and demethylation of tumor suppressor genes, in response to RSV was described in another study in human breast cancer cells [17]. Medina-Aguilar et al. showed that differentially methylated genes were involved in cellular oncogenic pathways, including Wnt, MAPK, and JAK/STAT. A subset of oncogenes was hypermethylated upon treatment with RSV, including *AKT1*, *STAT3*, and *NOTCH2* [17], which were enriched with pathways identified in our genome-wide study as well [16]. Among the top hypermethylated RSV targets, we found upstream activators of several key signaling pathways, and the highest changes were detected for *GLI2*-, a transcriptional activator of Hedgehog signaling, and *WNT4*-, a Wnt signaling ligand. Pathways controlled by the two genes, Hedgehog and Wnt, have canonical and non-canonical signaling routes and play essential roles in normal breast stem cell function and mammary gland development during embryogenesis, postnatal development, homeostasis, and pregnancy [18,19,20,21,22]. However, once aberrantly overactive, the pathways become drivers of mammary epithelial cell transformation, cell invasion, and metastasis [18,19,20,21,22]. In breast cancer, Wnt signaling drives cell proliferation and metastasis and was recently shown to suppress the anti-tumor immune response [20], for example, through its major target, Myc, that activates expression of cell antigens (e.g., *CD47*), preventing tumor cell recognition by the immune cells [23]. The Wnt pathway also activates a variety of genes that facilitate stemness maintenance (e.g., by increasing the expression of *CD44* antigen characteristic of breast cancer stem cells [24]) and drug resistance (e.g., by activating MDR1 transporter protein [25]). The importance of Wnt signaling specifically in breast cancer is further reflected in phenotype shaping and the classification of breast cancer [20]. For example, invasive lobular carcinoma is characterized by the loss of *β-catenin* expression, whereas invasive ductal carcinoma expresses *β-catenin* and has a poorer prognosis than the lobular type. As Wnt signaling, the Hedgehog pathway is implicated in breast cancer stem cell functioning and increases MDR1 transporter protein in cancer stem cells, leading to drug resistance [26]. Through GLI1, which acts as a transcription factor, Hedgehog signaling leads to increased expression of *CXCR4* receptors that are linked to enhanced metastatic properties and breast cancer cell survival [27]. While the mechanisms are still under investigation, the appearance of cancer-associated fibroblasts in the tumor microenvironment is accompanied by the activation of Hedgehog signaling and the increase in the number of breast cancer stem cells [28].

More importantly, crosstalk occurs between these two pathways in cancer due to the ability of Hedgehog signaling proteins to regulate the activity of the Wnt pathway, and vice versa [29,30,31,32,33]. This interdependent regulation allows for cancer therapies to mutually target the activity of both pathways. Interestingly, dietary polyphenols, specifically RSV, have been shown to suppress the Hedgehog and Wnt signals in breast cancer [17,34,35]. For instance, RSV was shown to induce autophagy in breast cancer cells in vitro and in xenograft tumors, and thus suppress cancer stem cells via inhibition of the Wnt/β-catenin pathway [36]. Importantly, these effects of RSV were significantly reduced upon the overexpression of *β-catenin*, which delivers mechanistic evidence for the role of Wnt signaling in RSV effects on autophagy and stemness in breast cancer [36]. When combined with anti-cancer agents (e.g., salinomycin), RSV synergistically induced apoptosis of ER-positive cells through the inhibition of Wnt signaling [35]. With regard to the Hedgehog pathway, RSV effects have not been explored specifically in breast cancer. However, RSV was demonstrated to inhibit Hedgehog signaling in pancreatic, gastric, and cervical cancers, which was accompanied by increased apoptosis and decreased cell proliferation, invasion, and metastasis [37,38,39,40].

Despite a growing body of evidence, mechanisms of these RSV-mediated effects on the Hedgehog and Wnt pathways and their crosstalk are scarcely explored. Our genome-wide investigation suggests that polyphenols, specifically RSV, may act through DNA methylation to regulate the oncogenic signaling pathways. However, it remains unclear whether RSV-mediated increases in the DNA methylation at regulatory regions of upstream regulators of the pathways, *GLI2* and *WNT4*, have any potential functional effects on the gene transcriptional activity and signal transduction. In the present study, we, therefore, test the impact of RSV-mediated DNA hypermethylation at those upstream activators on chromatin condensation, gene transcription, and transcription factor occupancy, followed by investigating pathways’ activities.

## 2. Materials and Methods

### 2.1. Cell Culture and Treatment

A human MCF10CA1a breast cancer cell line was cultured in DMEM/F12 (1:1) medium (Gibco, Thermo Fisher Scientific, Waltham, MA, USA) supplemented with 5% horse serum (Gibco, Thermo Fisher Scientific, Waltham, MA, USA), 1 U/mL penicillin, and 1 µg/mL streptomycin (Gibco, Thermo Fisher Scientific, Waltham, MA, USA). The MCF10CA1a cell line was obtained from Dr. Dorothy Teegarden, Purdue University, IN, USA, and represents poorly differentiated tumors with a high invasive capacity. The cell line was derived from tumor xenografts of MCF10A cells transformed with constitutively active Harvey-ras oncogene. The human MCF-7 breast cancer cell line (#HTB-22, ATCC, Manassas, VA, USA) was maintained in MEM (1:1) medium (Gibco, Thermo Fisher Scientific, Waltham, MA, USA) supplemented with 0.01 mg/mL insulin (Sigma, St. Louis, MO, USA), 10% fetal bovine serum (Gibco, Thermo Fisher Scientific, Waltham, MA, USA), 1 U/mL penicillin, and 1 µg/mL streptomycin (Gibco, Thermo Fisher Scientific, Waltham, MA, USA). Resveratrol (RSV, Sigma, St. Louis, MO, USA) was resuspended in ethanol and 10 mM stock solution was stored at −20 °C. Cells were grown in a humidified atmosphere of 5% carbon dioxide at 37 °C. A day prior to treatment with RSV at different concentrations, cells were plated at a density of 3 × 10^5^ per 10 cm tissue culture dish. After 4 days, cells were split 1:50, allowed to attach overnight, and exposed to RSV for an additional 4 days (overall, 9-day treatment). The number of viable and dead cells was determined using a trypan blue exclusion test at 4-day and 9-day time points. The IC_50_ dose, defined as a concentration that reduces cell growth by 50% and leads to not more than 10% of dead cells compared to vehicle-treated cells (ethanol), was established at both time points of exposure to RSV.

### 2.2. DNA Isolation and Pyrosequencing

Standard phenol/chloroform extraction protocol was used for DNA isolation. DNA bisulfite conversion was performed as previously described and amplified with biotinylated primers specific for tested gene regulatory regions using HotStar Taq DNA polymerase (Qiagen, Germantown, MD, USA) (please see Supplementary Materials, Table S1, for primer sequences) [16]. Pyrosequencing of the biotinylated DNA strands was performed in the PyroMark Q48 Autoprep instrument (Qiagen, Germantown, MD, USA), as previously described [41]. Quantification of the percentage of methylation at a single CpG site resolution was performed using PyroMark Q48 software (version 4.3.3).

### 2.3. RNA Isolation, cDNA Synthesis, and qRT-PCR

Isolation of total RNA was performed with the TRIzol reagent (Invitrogen, Thermo Fisher Scientific, Waltham, MA, USA), according to the manufacturer’s protocol. AMV reverse transcriptase (20 U, Roche Diagnostics, Indianapolis, IN, USA) was used to synthesize cDNA from 1 μg of total RNA, as recommended by the manufacturer. The quantitative real-time PCR reaction was carried out in a LightCycler 480 machine (Roche, Indianapolis, IN, USA) using 2 µL of cDNA (33 ng), 400 nM forward and reverse primers listed in Supplementary Table S1, and 10 µL of LightCycler 480 SybrGreen I Master (Roche Diagnostics, Indianapolis, IN, USA) in a final volume of 20 µL. Amplification conditions were as follows: denaturation at 95 °C for 10 min, amplification for 60 cycles at 95 °C for 10 s, annealing temperature for 10 s, 72 °C for 10 s, and final extension at 72 °C for 10 min. Quantification was performed using a standard curve and analyzed using LightCycler 480 software (version 1.5.1). Relative gene expression levels of target genes are presented as genes of interest/GAPDH (reference gene).

### 2.4. Chromatin Immunoprecipitation (ChIP) and Quantitative ChIP (qChIP)

Chromatin immunoprecipitation (ChIP) was performed to determine DNA–protein interactions, as previously described [16,42]. We used the following primary antibodies: anti-trimethyl-Histone H3 lysine 27 rabbit antibody (H3K27me3, #07-449, MilliporeSigma, Oakville, ON, Canada), anti-acetyl-Histone H3 lysine 27 rabbit antibody (H3K27ac, #07-360, MilliporeSigma, Oakville, ON, Canada), anti-acetyl-Histone H3 lysine 9 rabbit antibody (H3K9ac, #07-352, MilliporeSigma, Oakville, ON, Canada), and anti-OCT1 mouse antibody (#MAB5434, MilliporeSigma, Oakville, ON, Canada). ChIP DNA was used as a template for qPCR (quantitative ChIP, qChIP) where 25 ng of input, antibody-bound, and IgG-bound DNA was used as starting material in all conditions. The levels of occupancy of the proteins were expressed as (Bound-IgG)/Input.

### 2.5. Phospho-Antibody Wnt Signaling Array

Wnt Signaling Phospho-antibody Array and Antibody Array Assay Kit (Full Moon Biosystems, Sunnyvale, CA, USA) were used to determine the phosphorylation levels of the key Wnt signaling mediators. Alexa Flor 555 Conjugate Streptavidin (Thermo Fisher Scientific, Waltham, MA, USA) was used as a dye for detection. Array slides were scanned to detect florescence intensity using the Agilent G4900DA SureScan Microarray Scanner System (Agilent, Santa Clara, CA, USA). Array images were analyzed using ImageJ software (version 1.50) for florescence quantification and florescence intensity was normalized to the blank signal.

### 2.6. Statistical Analysis

An unpaired *t*-test with a two-tailed distribution was used for the statistical analysis of the data. Each value represents the mean ± S.D. of three independent experiments (biological replicates). The results were considered statistically significant when *p* < 0.05.

## 3. Results

### 3.1. Genes, That Are Hypermethylated upon Exposure to Resveratrol (RSV), Are Associated with Numerous Oncogenic Signaling Pathways

RSV treatment led to a decrease in cell growth rate in a dose-dependent manner in both human non-invasive MCF-7 and highly invasive MCF10CA1a breast cancer cells. The concentration of 15 µM was determined as the IC_50_ concentration for a 9-day treatment in MCF10CA1a [16,43] and MCF-7 cells (Figure S1). The IC_50_ value was higher on day 4 but remained comparable in both cell lines, at approximately 21*–*22 µM (Figure S1). Considering the evidence of non-linear dose–response for RSV chemopreventive effects and the higher efficacy of lower vs. higher RSV doses in cancer chemoprevention [44], our goal was to select the lowest dose possible for further experiments on RSV effects. As the same effect, namely 50% cell growth inhibition, was achieved at a lower RSV dose at a 9-day time point, we proceeded with the 9-day 15 µM RSV treatment in further experiments.

**Figure 1:**
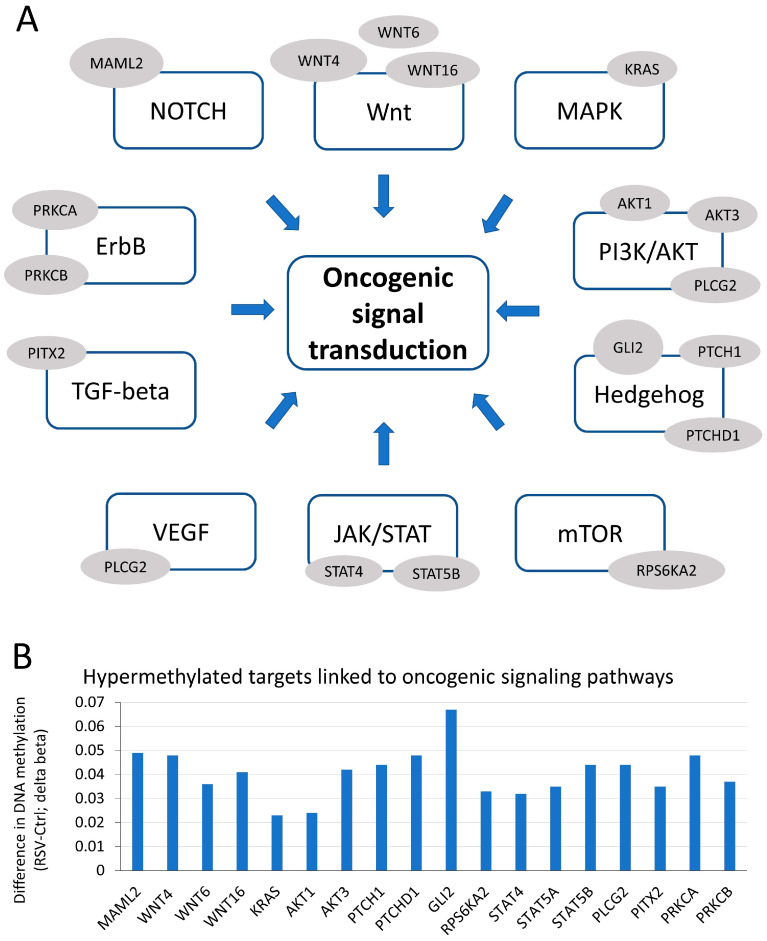
Genome-wide DNA methylation profiling identifies hypermethylated genes that are linked to regulation of multiple oncogenic signaling pathways in response to resveratrol (RSV) in breast cancer cells. (**A**) A scheme depicting oncogenic signaling pathways with their upstream regulators that are hypermethylated upon RSV exposure. (**B**) Differences in DNA methylation between 15 µM RSV-treated and vehicle-treated MCF10CA1a breast cancer cells, as determined by Infinium HumanMethylation 450 K BeadChIP microarray. The difference is expressed as delta beta, which corresponds to the difference in bead intensities (beta values). The data were extracted from GSE80794 from our previous work [16].

In a study previously conducted by our group, we performed an Illumina 450 K methylation array and identified alterations in the DNA methylation landscape in human invasive MCF10CA1a breast cancer cell lines in response to a 9-day treatment with 15 µM RSV [16]. Herein, exploring this dataset (GSE80794) and applying bioinformatic tools such as Gene Ontology, KEGG, and the DAVID knowledgebase, we have found that RSV-hypermethylated targets are enriched with key oncogenic signal transduction pathways, including Notch, Wnt, Hedgehog, TGF-β, MAPK, and AKT (Figure 1A). These pathways are well established to drive cancer development [45]. RSV-hypermethylated targets, which are classified within a specific functional category of a given signaling pathway and distinguished by the most significant increase in DNA methylation following RSV treatment compared to the control (Ctrl; cells treated with vehicle-ethanol), are illustrated in Figure 1A. The differences in the DNA methylation levels, expressed as delta beta values, are plotted in Figure 1B. One of the highest levels of hypermethylation was detected for *GLI2* and *WNT4* from the Hedgehog and Wnt signal transduction pathways, respectively. Of importance, these two genes are upstream components activating their respective pathway and creating crosstalk between the two pathways [29].

### 3.2. GLI2 and WNT4 from Hedgehog and Wnt Signaling Pathways, Respectively, Are Hypermethylated and Downregulated in Response to Resveratrol (RSV)

**Figure 2:**
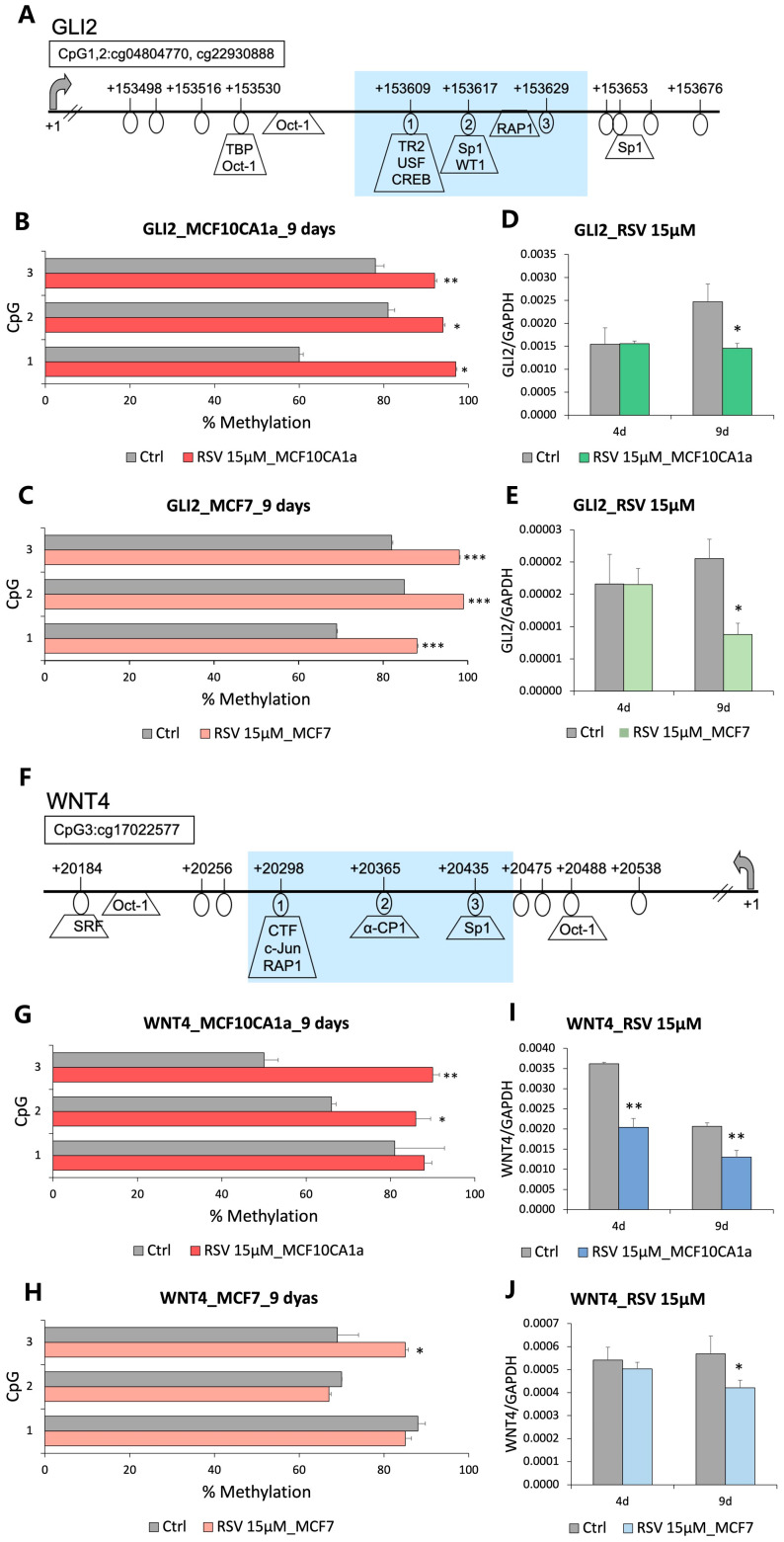
Hypermethylation and downregulation of GLI2 and WNT4 in response to resveratrol (RSV) in breast cancer cells. (**A**,**F**) A map of the GLI2 (**A**) and WNT4 (**F**) enhancer region, where the light blue shaded area represents the entire fragment tested by pyrosequencing and quantitative chromatin immunoprecipitation (qChIP). Transcription start site (TSS) is indicated by +1, transcription factors were predicted using Transfac, and pyrosequenced CpG sites are circled and numbered. CpG covered on Illumina microarray platform is indicated above the gene map along with CpG loci identifier number (so-called cluster CG#). (**B**,**C**) Average methylation status of CpG sites in GLI2, from the Hedgehog signaling pathway, in invasive MCF10CA1a (**B**) and non-invasive MCF-7 (**C**) breast cancer cells. (**D**,**E**) Expression of GLI2 upon 4-day and 9-day treatment of invasive MCF10CA1a (**D**) and non-invasive MCF-7 (**E**) breast cancer cells with 15 µM RSV, as determined by qRT-PCR. (**G**,**H**) Average methylation status of CpG sites in WNT4, from the Wnt pathway, in invasive MCF10CA1a (**G**) and non-invasive MCF-7 (**H**) breast cancer cells upon 9-day exposure to 15 µM RSV. (**I**,**J**) Expression of WNT4 upon 4-day and 9-day treatment of invasive MCF10CA1a (**I**) and non-invasive MCF-7 (**J**) breast cancer cells with 15 µM RSV. All results represent mean ± SD of three independent experiments; *** *p* < 0.001, ** *p* < 0.01, * *p* < 0.05.

We further used pyrosequencing to quantify the DNA methylation changes within enhancers of *GLI2* and *WNT4* in MCF10CA1a and MCF-7 cells treated with 15 µM of RSV for 9 days (Figure 2A,F). Within three CpG sites in *GLI2* enhancer, we detected a 15*–*38% increase in DNA methylation upon RSV in the invasive ER(−) MCF10CA1a cell line (Figure 2B). Similar effects with a 15*–*20% increase in DNA methylation were observed in non-invasive ER(+) MCF-7 breast cancer cells (Figure 2C). *GLI2* hypermethylation was linked to gene downregulation by 40% in MCF10CA1a cells (Figure 2D) and by 57% in MCF-7 cells (Figure 2E). An inverse correlation between DNA methylation of an enhancer region and gene expression was also identified for the *WNT4* oncogene. Across CpG sites 2 and 3, we detected a 20*–*40% increase in DNA methylation (Figure 2G), which was accompanied by a 30% drop in *WNT4* expression in MCF10CA1a treated with RSV (Figure 2I). In non-invasive MCF-7 cells, a 30% downregulation of *WNT4* (Figure 2J) was associated with a 15% hypermethylation at CpG2 (Figure 2H). Taken together, we report that *GLI2* and *WNT4* are hypermethylated and downregulated in response to 15 µM RSV in MCF10CA1a and MCF-7 cells. Of note, *WNT4* was downregulated already at a 4-day treatment in MCF10CA1a cells, which could possibly imply that *WNT4* is an early response gene in RSV-mediated epigenetic effects (Figure 2I).

### 3.3. Genes Downstream of the Hedgehog and Wnt Signaling Are Downregulated in Response to Resveratrol (RSV)

**Figure 3:**
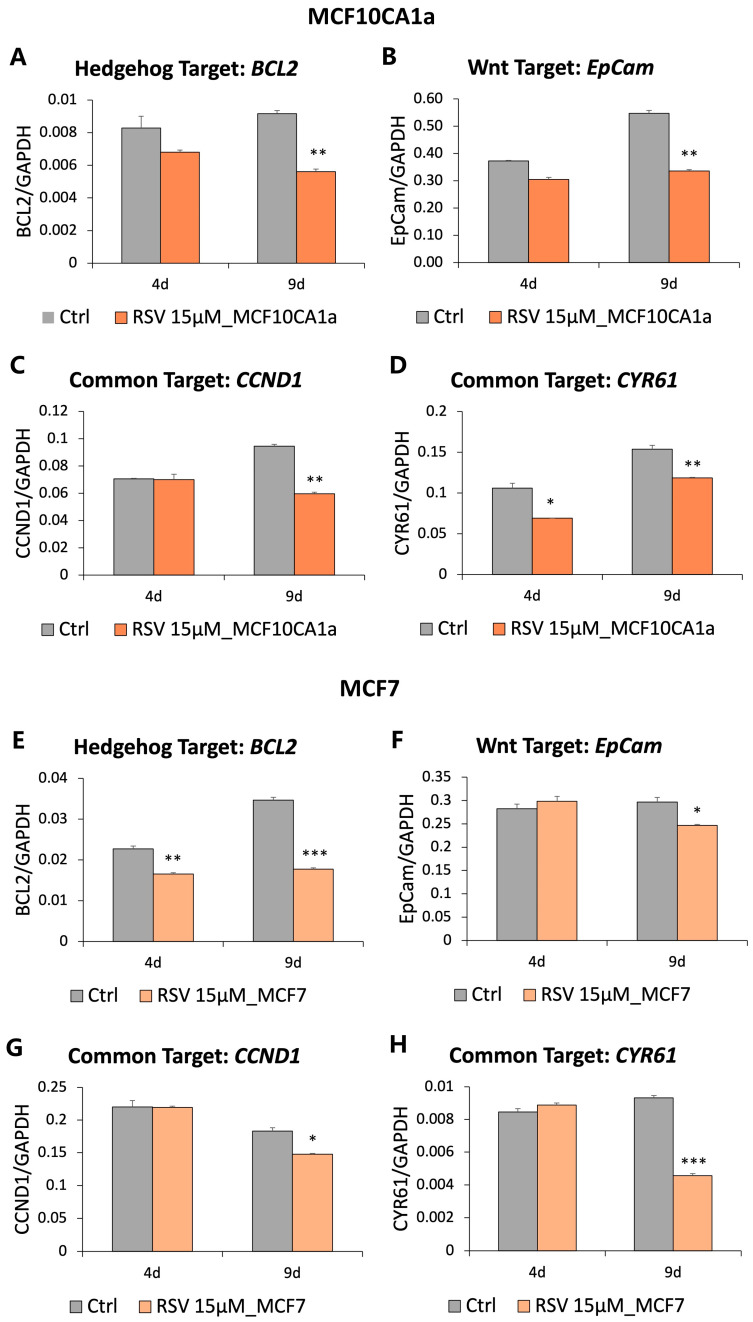
Expression of genes downstream of the Hedgehog and Wnt signaling pathways in response to resveratrol (RSV) in breast cancer cells. (**A**,**B**,**E**,**F**) Expression of BCL2 (**A**,**E**) and EpCam (**B**,**F**), the Hedgehog and Wnt signaling target genes, respectively, as quantified by qRT-PCR in MCF10CA1a (**A**,**B**) and MCF-7 (**E**,**F**) breast cancer cells following 4 and 9 days of 15 µM RSV treatment. (**C**,**D**,**G**,**H**) Expression of CCND1 (**C**,**G**) and CYR61 (**D**,**H**), target genes of both the Hedgehog and Wnt pathways, as quantified by qRT-PCR in MCF10CA1a (**C**,**D**) and MCF-7 (**G**,**H**) breast cancer cells following 4 and 9 days of 15 µM RSV treatment. All results represent mean ± SD of three independent experiments; *** *p* < 0.001, ** *p* < 0.01, * *p* < 0.05.

In order to validate that the downregulation of components upstream of the Hedgehog and Wnt pathways in response to RSV is biologically significant and results in the inhibition of the oncogenic pathways, we measured the expression of genes downstream of both pathways (Figure 3). In response to 15 µM RSV treatment over the course of 9 days in the highly invasive MCF10CA1a breast cancer cells, we observed statistically significant downregulation of a Wnt signaling gene target, *EpCam* (39% decrease), a Hedgehog signaling gene target, *BCL2* (39% decrease), and two common gene targets of both the Wnt and Hedgehog pathways, *CCND1* (37% decrease) and *CYR61* (23% decrease) (Figure 3A–D). Similar to MCF10CA1a, downstream targets of the pathways were reduced in RSV-treated non-invasive MCF-7 cells, with close to a 20% decrease in expression of *EpCam* and *CCND1*, and a 50% drop in *CYR61* and *BCL2* (Figure 3E–H). Altogether, our results suggest that the epigenetic silencing of *WNT4* and *GLI2* induced by RSV treatment leads to attenuation of the Wnt and Hedgehog signal transduction, as evidenced by decreased transcription of target genes of the pathways. Interestingly, treatment with 15 µM RSV over 9 days was capable of selectively inhibiting cancer cell growth by 50% in MCF10CA1a [16] and in MCF-7 cells (Figure S1), whilst having negligible effects on normal mammary epithelial cells [16]. This suggests that cancer cell growth inhibition by RSV may possibly be partially explained through downregulation of the oncogenic pro-proliferative Wnt and Hedgehog signaling.

### 3.4. Compacted Chromatin Structure and Decreased OCT1 Transcription Factor Occupancy at Regulatory Regions of GLI2 and WNT4 in Response to Resveratrol (RSV)

It is well documented that epigenetic mechanisms of gene regulation possess a great deal of crosstalk between each other, including DNA methylation and histone modifications [46,47,48]. On the one hand, trimethylation of histone H3 at lysine 27 (H3K27me3) is generally associated with the formation of inaccessible/closed chromatin (heterochromatin) and ultimately gene repression [49]. On the other hand, acetylation of H3 at lysine 9/27 (H3K9ac/H3K27ac) are active enhancer marks and are typically associated with increased gene transcription and a more open chromatin state (euchromatin) [50,51].

**Figure 4:**
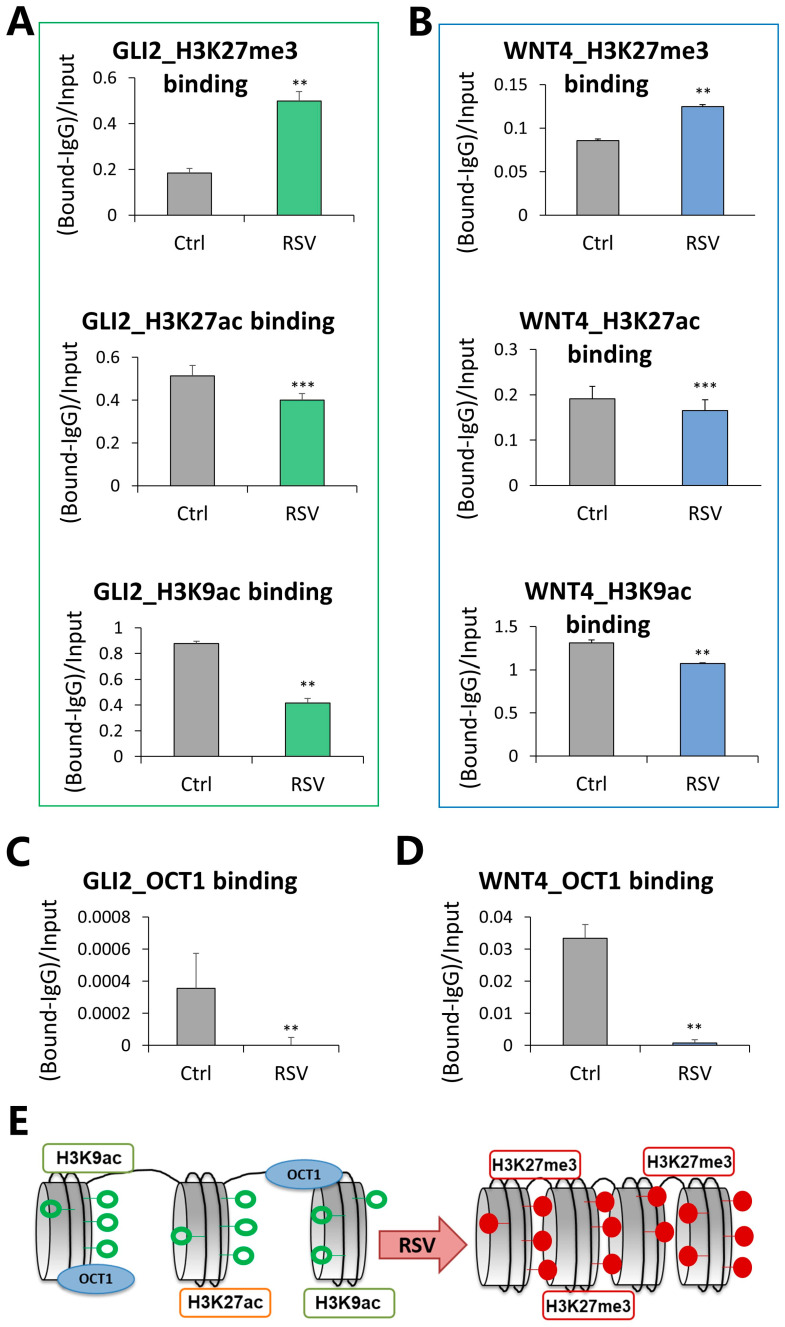
Chromatin accessibility and transcription factor binding at the enhancer regions of GLI2 and WNT4 in response to resveratrol (RSV). (**A**,**B**) Occupancy of trimethylation of lysine 27 of histone H3 (H3K27me3), a histone repressive mark, as well as acetylation of lysine 27 and 9 of histone H3 (H3K27ac and H3K9ac), active histone marks, at the GLI2 (**A**) and WNT4 (**B**) enhancer regions in MCF10CA1a breast cancer cells following 9-day exposure to 15 µM RSV. (**C**,**D**) Binding of OCT1, a transcription factor associated with activating genes linked to cancer promotion, at the GLI2 (**C**) and WNT4 (**D**) enhancer regions in MCF10CA1a breast cancer cells upon 9-day exposure to 15 µM RSV. (**E**) A scheme depicting a change from open chromatin to condensed chromatin packaging in response to RSV. All results represent mean ± SD of three independent experiments; *** *p* < 0.001, ** *p* < 0.01.

DNA hypermethylation at *GLI2* and *WNT4* enhancers and reduced mRNA levels of *GLI2* and *WNT4* genes indicate a closed chromatin structure at those regulatory regions. To assess chromatin, we measured repressive and active histone marks, including H3K27 tri-methylation, and H3K27 and H3K9 acetylation, in response to MCF10CA1a cells to a 9-day treatment with 15 µM RSV (Figure 4). *GLI2* enhancer was 2.7 times more enriched with a repressive mark, H3K27me3, simultaneously losing active marks, namely 22% of H3K27ac and 53% of H3K9ac occupancy (Figure 4A). Within the *WNT4* enhancer, we detected a 1.5-fold increase in H3K27me3 binding, which was accompanied by a 14% decrease in H3K27ac and an 18% decrease in H3K9ac occupancy (Figure 4B).

Importantly, our previous studies demonstrated that 80% of RSV hypermethylated target genes contain a sequence corresponding to the OCT1 response element, which we quantitatively validated for a set of genes [14,15,16]. Using chromatin immunoprecipitation (ChIP), we experimentally confirmed OCT1 binding to *GLI2* and *WNT4* enhancer regions in control cells (Figure 4C*,*D). The compacted chromatin structure at both *GLI2* and *WNT4* enhancers, as indicated by histone marks (Figure 4A*,*B) and DNA methylation (Figure 2), was associated with a robust almost 98% decrease in OCT1 transcription factor binding upon RSV treatment (Figure 4C,D).

### 3.5. The Wnt Pathway Activity Is Attenuated as Reflected by Changes in Protein Phosphorylation upon Exposure to Resveratrol (RSV)

The post-translational modifications of proteins play an important role in signal transduction pathways as these modifications significantly affect the structure and functioning of upstream signaling mediators [52,53]. Furthermore, it has been previously demonstrated that numerous mediators of Wnt signaling (e.g., β-catenin, APC, GSK3) are regulated by phosphorylation, which ultimately affects the activity of the Wnt signaling pathway [54,55], and, consequently, other pathways Wnt signaling is interconnected with (e.g., Hedgehog) [29,30,33]. We therefore asked a question whether RSV-mediated epigenetic silencing of *WNT4* and *GLI2* is accompanied by an overall decrease in oncogenic signal transduction. Utilizing an ELISA-based phosphorylation array, we qualitatively determined the phosphorylation levels of proteins within the Wnt pathway.

**Figure 5:**
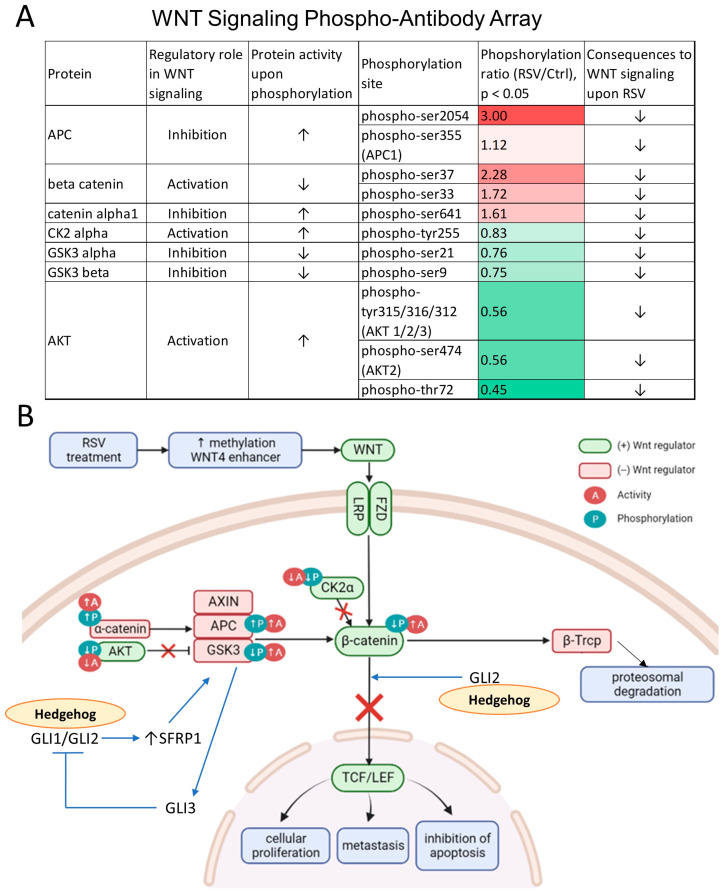
The activity of Wnt signaling mediators as measured based on the level of protein phosphorylation in response to resveratrol (RSV). (**A**) Protein phosphorylation ratio between MCF10CA1a breast cancer cells treated with resveratrol (RSV) and treated with vehicle (ethanol, Ctrl). Wnt Signaling Phospho-antibody Array and Antibody Array Assay Kit were used to determine phosphorylation levels of key mediators of the Wnt pathway. Streptavidin covalently attached to a fluorescent label was used as dye for detection. The florescence intensity was analyzed using ImageJ. Changes in protein activity were quantified using antibodies for the phosphorylated and unphosphorylated sites in each protein. Values of the phosphorylation ratio are color-coded with red and green colors corresponding, respectively, to the increase or decrease in phosphorylation levels of a given protein in response to RSV as compared with control cells (RSV/Ctrl). All results represent mean ± SD of three independent experiments. (**B**) A scheme of the Wnt pathway with indicated changes in the activity and phosphorylation levels of the key mediators. Up and down arrows correspond, respectively, to the increase or decrease in the activity or phosphorylation of given proteins. A cross through symbol reflects the inhibition of a given route due to changes in the activity/phosphorylation of an upstream regulator in response to RSV. The major regulatory routes in the interplay between the Wnt and Hedgehog pathways are indicated with blue arrows/lines. Upon activation by GLIs, SFRP1 activates GSK3-beta, which phosphorylates beta-catenin decreasing its activity. GLI2 can promote nuclear localization of beta-catenin, thereby increasing Wnt signal transduction. GSK3-beta-mediated phosphorylation of GLI3 inhibits GLI1/GLI2 activity and thus the Hedgehog pathway. Biorender was used to create the scheme.

Phosphorylation levels of APC and catenin alpha 1, which are Wnt pathway-inhibiting proteins, increased in response to RSV treatment indicating an increased activity of APC and catenin alpha 1 (Figure 5A). In contrast, non-phosphorylated beta-catenin is a strong activator of the Wnt signaling. Its activity was decreased by RSV, as reflected in increased phosphorylation levels at serine positions Ser33 and Ser37 [54]. Phosphorylated beta-catenin is directed into ubiquitination and proteasomal degradation rather than to stimulation of TCF/LEF-mediated transcription (Figure 5B) [54]. On the other hand, exposure of MCF10CA1a cells to RSV resulted in decreased phosphorylation and thus impaired activity of two activators of Wnt signaling, namely AKT and CK2-alpha (Figure 5A). Phosphorylation levels of GSK3 were also decreased upon RSV treatment (Figure 5A), which could potentially be explained by suppression of AKT that catalyzes phosphorylation and consequently inhibition of GSK3 (Figure 5B). Active GSK3, in turn, phosphorylates APC and beta-catenin, which leads to an increase or decrease in the activity of these targets, respectively, altogether attenuating the Wnt pathway (Figure 5B). Collectively, changes in phosphorylation levels of proteins from the Wnt pathway lead to the inhibition of major routes (marked in red in Figure 5B), which turns off the oncogenic signal transduction.

## 4. Discussion

One of the mechanisms by which oncogenic growth occurs in breast cancer is through a variety of established signaling pathways including the Notch, Wnt, Hedgehog, TGF-beta, MAPK, and AKT pathways [45]. Each pathway consists of a cascade of signaling molecules resulting in an aspect of development or growth that is crucial during the early developmental stages of life and silenced following the normal stages of growth and development. In cancer, these pathways are reactivated in adult tissues to levels not normally observed, resulting in abnormal cellular growth, tumor formation, and cancer initiation [20,22]. Moreover, the Hedgehog and Wnt oncogenic pathways constitute key stemness-associated pathways facilitating self-renewal and cancer stem cell-induced tumor initiation and progression in different types of cancer, including breast cancer [45]. Of importance, the co-activation of Hedgehog and Wnt signals has been associated with early recurrence, decreased survival, and overall poorer prognosis in triple-negative breast cancer patients [33].

RSV has been previously shown to inhibit oncogenic signaling pathways, including Wnt and Hedgehog in different cancers, including breast cancer [34]. However, the mechanisms of RSV effects remain unexplained. As we and others previously reported, genes targeted for re-methylation and silencing by RSV in breast cancer cells are enriched with oncogenic functions and pathways (Figure 1), which might explain RSV-mediated decrease in cell growth and invasive properties [14,15,16,17]. Furthermore, numerous other bioactive compounds, particularly from a class of polyphenols, have been demonstrated to impact the DNA methylation patterns and other components of the epigenome [56,57,58]. The latest results from the randomized clinical trial, DIRECT PLUS study (NCT03020186), show that the green Mediterranean high polyphenol diet, rich in green tea and Mankai, shapes the methylome (DNA methylation landscape) and specifically affects components of the one-carbon metabolism that is one of the key pathways regulating the epigenetic modifications [57]. This recent finding is especially important as it validates polyphenols’ epigenetic effects in humans.

Of interest, among RSV-hypermethylated targets in MCF10CA1a breast cancer cells, a gene with the highest magnitude of change, *GLI2* (Figure 1B), is a transcriptional co-activator of Hedgehog signaling, which crosstalks with the Wnt pathway, for example, through the inhibitory action of GSK3-beta on both GLI and beta-catenin [29]. Because DNA hypermethylation was the highest at *WNT4* among identified hypermethylated WNTs, and WNT4 is a Wnt ligand shown to be particularly upregulated in breast cancer [59] and in subsets of breast cancer expressing autocrine human growth hormone (hGH) that drives transformation [60], we proceeded with the investigation of *GLI2* and *WNT4*. Another central aspect in our current study was breast cancer cells with diverse profiles of hormonal expression, ER-positive MCF-7 and triple-negative MCF10CA1a, to address the heterogeneity of breast cancer which is a major challenge in clinical settings.

Alterations in DNA methylation patterns in regulatory regions of *GLI2* and *WNT4* following a 9-day treatment with 15 μM RSV were initially identified using the Illumina 450K DNA methylation microarray in highly invasive MCF10CA1a cells [16] (Figure 1). Further quantitative analysis with pyrosequencing revealed significant loci-specific increases in DNA methylation in the enhancer regions of both genes in MCF10CA1a cells, as well as in non-invasive MCF-7 cells (Figure 2). Increases in DNA methylation patterns in regulatory regions of a gene, such as an enhancer, are consistent with the inhibition of transcriptional initiation and therefore a decrease in expression of the gene [14,61]. Moreover, we previously demonstrated that stilbenoid polyphenols specifically target enhancer regions for epigenetic silencing in breast cancer cells [14]. Using qRT-PCR, we validated this proposed downregulation with the experimental detection of decreases in *GLI2* and *WNT4* mRNA levels following 9 days of 15 μM RSV treatment in MCF-7 and MCF10CA1a breast cancer cells (Figure 2). Interestingly, the expression of *GLI2* and *WNT4* was not affected at a 4-day time point, implying that the tested targets are late-response genes. *WNT4* in MCF10CA1a cells was an exception, potentially indicating the existence of an alternative mechanism of transcriptional regulation of *WNT4* at a 4-day time point and its role in early response to RSV. As epigenetic components cooperate to regulate gene transcription in mammalian cells, we measured histone protein marks to further elucidate changes in chromatin condensation and transcriptional activity of the regions [46,62]. Increased binding of H3K27me3 repressive mark and decreased H3K27ac and H3K9ac active marks were observed, correlating with the detected increases in DNA methylation and gene silencing following RSV treatment (Figure 4). Our findings indicate that RSV promotes the formation of condensed chromatin (heterochromatin) in the regulatory regions of *GLI2* and *WNT4* (Figure 4E). H3K27ac has been shown to specifically mark active enhancers [50], and thus its decrease would indicate lower enhancer activity. Of note, a strong crosstalk between DNA methylation and histone methylation has been established when studying DNA methylation maintenance during DNA replication [62]. Furthermore, DNMTs, including de novo DNMT3A and DNMT3B, interact with histone tails and pre-existing histone methylation has been shown to participate in the recruitment of DNMTs and the distribution of DNA methylation in the genome [62]. However, the interplay between DNA and histone methylation may depend on the position of histone methylation, as exampled by H3K27me3. Genome-wide studies show that DNA methylation and H3K27me3 are mutually exclusive in stem cells; however, the two marks can co-exist in low CpG-dense regions in more differentiated cells [62]. Importantly, enhancer regions are most often characterized by a low level of CpG density, which suggests a co-existence of DNA methylation and H3K27me3 in mammary epithelial cells—our experimental model.

Increased H3K27me3 occupancy at the target genes in response to RSV could also be facilitated via the Hedgehog pathway-dependent mechanism. Recent evidence indicates an intriguing mechanism of regulation of H3K27me3 occupancy via the Hedgehog pathway in breast cancer. It was shown that GLI1 from Hedgehog signaling recruits JMJD3 histone demethylase to specific DNA loci, which results in H3K27 demethylation and consequently activation of oncogenes [63]. Hence, the suppression of Hedgehog signaling upon RSV would interfere with JMJD3 binding and thereby facilitate H3K27 trimethylation and oncogene inhibition.

Epigenetic downregulation of *GLI2* and *WNT4*, which are members of breast cancer signaling pathways driving cell proliferation, differentiation, and oncogenesis, would be expected to decrease stimulation of the Hedgehog and Wnt pathways, in part justifying the decreased cell proliferation observed following RSV treatment in MCF10CA1a [16] and MCF-7 cells (Figure S1). Hence, we have investigated whether the inhibitory signal is transferred downstream to the pathway target genes by measuring their mRNA levels. Indeed, we have observed a consistent decrease in the expression of all selected target genes, which included *BCL2* as the Hedgehog target, *EpCAM* as the Wnt target, and *CCND1* and *CYR61* as targets common between the two pathways (Figure 3). Importantly, epigenetic regulation of the Hedgehog and Wnt pathways has been previously reported in multiple cancers [64,65]. For instance, the upregulation of *WNT2* in colorectal cancer was mechanistically linked to a decrease in repressive histone mark H3K27me3 [64]. *GLI1* was upregulated which coincided with loss of mono-methylation at histone 3 lysine 4, while *SHH* was hypomethylated and overexpressed in breast cancer [65]. On the contrary, increased expression of *GLI2* was linked to various microRNAs in numerous cancers, including breast cancer [65]. To our knowledge, epigenetic regulation of *WNT4* has not been previously reported however recent evidence indicates that WNT4 is secreted by colorectal tumors and promotes cancer progression, metastasis, and angiogenesis through the canonical Wnt signaling pathway [66].

Interestingly, there is a strong regulatory connection between the Wnt and Hedgehog pathways and this crosstalk has been shown to play an important role in cancer development and progression, including breast cancer [29,30,33]. Non-canonical Hedgehog signaling, which is PTCH1-dependent and SMO and GLI-independent, is believed to increase Wnt activity in the intestinal tissue [64]. On the other hand, several studies in colon cancer show that the canonical Hedgehog pathway inhibits Wnt by regulating the expression of *SFRP1* via its effector proteins, GLI1 and GLI2 [64]. Upon activation by GLIs, SFRP1 activates GSK3-beta which phosphorylates beta-catenin, abolishing the signal transfer to TCF/LEF for transcriptional stimulation of Wnt-responsive genes [30] (Figure 5B). The link between Hedgehog and Wnt seems to be even more complicated as the existing evidence also indicates that GLI2 can promote nuclear localization of beta-catenin, thereby increasing Wnt signal transduction and driving cell proliferation of osteosarcoma cells [64,67] (Figure 5B). With regard to the regulatory influence of Wnts on Hedgehog, the Wnt pathway has been shown to inhibit GLI-mediated transcriptional regulation of Hedgehog-responsive genes and thus the Hedgehog signal transduction [30]. This is achieved by GSK3-beta-mediated phosphorylation of GLI3 that subsequently inhibits GLI1/GLI2 activity [29] (Figure 5B). Similar to GSK3-beta, which regulates the activity of GLIs and beta-catenin, SUFU tumor suppressor, a well-known negative regulator of the Hedgehog pathway, inhibits both Hedgehog and Wnt through the interaction with GLIs and beta-catenin as well [29]. Of importance, the epigenetic upregulation of both pathways was demonstrated to occur through miRNA-150-mediated downregulation of SUFU in gastric cancer, further supporting the crosstalk between the pathways [68].

As Wnts and GSK3-beta-mediated protein phosphorylation appear to form one of the regulatory connections between the Hedgehog and Wnt signals, and the Wnt pathway is very well explored in terms of the activity–protein phosphorylation dependence, we further validated RSV-mediated oncogenic signal attenuation through a detailed investigation of Wnt signaling. We used a Wnt Signaling Phospho-antibody Array, demonstrating the activity of signaling pathway molecules based on the level of protein phosphorylation in MCF10CA1a cells after 9 days of 15 μM RSV treatment. Decreased activity was observed from key Wnt positive regulators including AKT, CK2-alpha, and beta-catenin. These key regulators are known to have a role in cancer promotion [45] and their activity was inhibited in cells following RSV treatment (Figure 5). On the other hand, increased activity was observed from Wnt negative regulators APC, GSK3, and alpha-catenin, which, consequently, has been linked in the literature with low Wnt signaling activity (Figure 5). Our model demonstrated an increase in the activity of these proteins, supporting the finding on the inhibition of Wnt signaling following RSV treatment. The alterations in signaling molecules of the Wnt pathway in response to RSV are consistent with beta-catenin degradation upon GSK3-beta-mediated phosphorylation resulting in a decline of the Wnt target gene transcriptional activity (Figure 5B). Simultaneously, GSK3-beta inhibits GLI proteins and, as a result, the Hedgehog pathway.

In our current study, polyphenols, specifically RSV, were shown to modify epigenetic marks and change the chromatin condensation in upstream genes positively regulating the Hedgehog and Wnt pathways, which was accompanied by inhibition of Hedgehog/Wnt common downstream targets and attenuation of oncogenic signal transduction. We established precise target genes that are affected by RSV providing potential targets for novel therapies. Although the in vitro controlled environment used in our study allows for a precise investigation of RSV targets, it makes it impossible to capture the effects of RSV metabolites. Other limitations include using only two cell lines and one single treatment schedule, which may not be translated into in vivo models and other study designs.

The future significance of RSV for cancer treatment is immense, particularly when considering usage as an adjunctive treatment to chemotherapy. Studies have begun to compare traditional chemotherapeutic treatments to combination treatments consisting of chemotherapeutics and RSV, as well as other polyphenols [35,58,69,70]. While the body of evidence demonstrating the effectiveness of these adjunctive treatments is not yet complete and in vivo data for these studies are growing, the implications of these combination therapies are robust, for example, potentiating the activity of doxorubicin and increasing sensitivity to tamoxifen and radiotherapy in breast cancer models [35]. The low-toxic properties of RSV result in increased treatment tolerability, and its anti-cancer effects may bring about additive treatment benefits [35,71]. Moreover, nanotechnological approaches are under investigation to increase RSV bioavailability and efficacy, including nanoparticles for targeted delivery of RSV in breast cancer [35,71]. Another beneficial aspect of RSV anti-cancer action, specifically the inhibition of oncogenic signaling pathways, could lie in overcoming resistance to known pathway inhibitors and chemotherapeutics. For instance, recent evidence shows the Wnt/beta-catenin pathway as a promoting factor in breast tumor drug resistance and the inhibition of the pathway by alkaloids indeed enhanced the efficacy of common chemotherapeutics in breast cancer [72]. This finding delivers the premise for RSV in reversing drug resistance by inhibiting the Wnt signal transduction and opens the door to further research.

Recent advances in gut microbiota and metabolomics, including the introduction of novel and more sensitive technologies (e.g., ultra-high-performance liquid chromatography coupled to a triple-quadrupole mass spectrometry system), unraveled a wide spectrum of RSV cellular and gut bacteria-derived metabolites [73,74]. Microbial metabolites, such as dihydro-resveratrol and lunularin, had even stronger anti-inflammatory and anti-cancer effects than RSV at in vivo relevant concentrations that were found in mouse tissues upon exposure to RSV [73]. These discoveries warrant further research on RSV derivatives and their role in cancer therapy, including their dose-dependent effects, pharmacological targets, and the possibility of pharmaceutical formulations.

When studying and applying RSV for cancer treatment, potential drug interactions must be considered as RSV and its metabolites have been shown to interact with drug-metabolizing enzymes and drug transporters, for example, cytochrome P450 (CYPs) [75]. Inhibiting important CYP enzymes and inducing conjugating enzymes are vital to RSV-mediated inhibition of carcinogen bioactivation and RSV-mediated elimination of toxic substances, respectively. However, as the same enzymes/pathways are involved in the metabolism of a variety of drugs, RSV might disrupt/potentiate their pharmacological action [75]. It is important to emphasize that these interactions matter when pharmacological concentrations of RSV are used; thereby, they might not constitute an obstacle when RSV-rich foods are consumed, as they contain low RSV concentrations. Importantly, growing evidence shows the high efficacy of low RSV doses in disease prevention [44], proving its bioactivity with a minimal possibility of nutrient-drug interactions.

## 5. Conclusions

Our findings demonstrate that RSV-mediated epigenetic alterations in transcriptional activity of oncogenes, that are located upstream of the Hedgehog and Wnt oncogenic signaling, are accompanied by the attenuation of the core oncogenic pathways. Of importance, the effects were detected in breast cancer cells with a different receptor status, triple negative and highly-invasive MCF10CA1a and non-invasive ER-positive MCF-7 cells. We provide scientific evidence that suggests dietary bioactive compounds as epigenetic agents that could combat epigenetic activation of genes driving breast cancer development and progression, and hence have the potential to be part of effective strategies in cancer prevention and/or treatment.

## Data Availability

The Illumina 450K methylation array data in MCF10CA1a cells upon 9-day exposure to 15 µM RSV are available from the Gene Expression Omnibus (accession numbers: GSE80794).

## Supplementary Materials

The following supporting information can be downloaded at: https://www.mdpi.com/article/10.3390/nu16050699/s1, Figure S1: resveratrol (RSV) exerts a dose-dependent effect on growth of breast cancer cells; Table S1: pyrosequencing, qRT-PCR, and quantitative ChIP (qChIP) primer sequences for the selected gene targets.

## Abbreviations

AKT1	AKT Serine/Threonine Kinase 1
AKT3	AKT Serine/Threonine Kinase 3
APC	APC Regulator of WNT Signaling Pathway
BCL2	BCL2 Apoptosis Regulator
CCND1	Cyclin D1
CK2α	Casein Kinase Two Alpha
CYR61	Cysteine-rich Angiogenic Inducer 61
EpCam	Epithelial Cell Adhesion Molecule
FZD	Frizzled Class Receptor
GAPDH	Glyceraldehyde-3-Phosphate Dehydrogenase
GLI	GLI Family Zinc Finger
GSK3α (GSK3-alpha)	Glycogen Synthase Kinase 3 Alpha
GSK3β (GSK3-beta)	Glycogen Synthase Kinase 3 Beta
JAK/STAT	Janus Kinase/Signal Transducers and Activators of Transcription
KRAS	KRAS Proto-oncogene, GTPase
LRP	LDL Receptor-related Protein
MAML2	Mastermind-like Transcriptional Coactivator 2
MAPK	Mitogen-activated Protein Kinase
NENF	Neudesin Neurotrophic Factor
NOTCH	Notch Receptor
NOTCH2	Notch Receptor 2
OCT1(SLC22A1)	Solute Carrier Family 22 Member 1
PITX2	Paired-like Homeodomain 2
PLCG2	Phospholipase C Gamma 2
PRKCA	Protein Kinase C Alpha
PRKCB	Protein Kinase C Beta
PTCH1	Patched 1
PTCHD1	Patched Domain Containing 1
PTEN	Phosphatase and Tensin Homolog
RARβ	Retinoic Acid Receptor Beta
RASAL2	RAS Protein Activator Like 2
RPS6KA2	Ribosomal Protein S6 Kinase A2
RSV	Resveratrol
SFRP1	Secreted Frizzled-related Protein 1
SMO	Smoothened, Frizzled Class Receptor
STAT3	Signal Transducer and Activator of Transcription 3
STAT4	Signal Transducer and Activator of Transcription 4
STAT5B	Signal Transducer and Activator of Transcription 5B
SUFU	SUFU Negative Regulator of Hedgehog Signaling
TCF/LEF	T-cell Factor/Lymphoid Enhancer Factor
TGF-β pathway	Transforming Growth Factor-beta
Wnt signaling	Wingless-related Integration Site
WNT16	Wnt Family Member 16
WNT4	Wnt Family Member 4
WNT6	Wnt Family Member 6
Β-Trcp (BTRC)	Beta-transducin Repeat Containing E3 Ubiquitin Protein

## References

[1] Siegel R.L., Miller K.D., Wagle N.S., Jemal A. (2023). Cancer statistics, 2023. CA Cancer J. Clin..

[2] Vietri M.T., D’Elia G., Benincasa G., Ferraro G., Caliendo G., Nicoletti G.F., Napoli C. (2021). DNA methylation and breast cancer: A way forward (Review). Int. J. Oncol..

[3] Roussos Torres E.T., Connolly R.M. (2023). Breast cancer epigenetics (Chapter 13). Epigenetic Cancer Therapy—Translational Epigenetics.

[4] Jenkins B.D., Rossi E., Pichardo C., Wooten W., Pichardo M., Tang W., Dorsey T.H., Ajao A., Hutchison R., Moubadder L. (2023). Neighborhood Deprivation and DNA Methylation and Expression of Cancer Genes in Breast Tumors. JAMA Netw. Open.

[5] Nishiyama A., Nakanishi M. (2021). Navigating the DNA methylation landscape of cancer. Trends Genet..

[6] Stefanska B., Salame P., Bednarek A., Fabianowska-Majewska K. (2012). Comparative effects of retinoic acid, vitamin D and resveratrol alone and in combination with adenosine analogues on methylation and expression of phosphatase and tensin homologue tumour suppressor gene in breast cancer cells. Br. J. Nutr..

[7] Wu Y., Kroller L., Miao B., Boekhoff H., Bauer A.S., Buchler M.W., Hackert T., Giese N.A., Taipale J., Hoheisel J.D. (2021). Promoter Hypermethylation Promotes the Binding of Transcription Factor NFATc1, Triggering Oncogenic Gene Activation in Pancreatic Cancer. Cancers.

[8] Stefanska B., Huang J., Bhattacharyya B., Suderman M., Hallett M., Han Z.G., Szyf M. (2011). Definition of the landscape of promoter DNA hypomethylation in liver cancer. Cancer Res..

[9] Stefanska B., Cheishvili D., Suderman M., Arakelian A., Huang J., Hallett M., Han Z.G., Al-Mahtab M., Akbar S.M., Khan W.A. (2014). Genome-wide study of hypomethylated and induced genes in patients with liver cancer unravels novel anticancer targets. Clin. Cancer Res..

[10] Mayol G., Martin-Subero J.I., Rios J., Queiros A., Kulis M., Sunol M., Esteller M., Gomez S., Garcia I., de Torres C. (2012). DNA hypomethylation affects cancer-related biological functions and genes relevant in neuroblastoma pathogenesis. PLoS ONE.

[11] Shukeir N., Stefanska B., Parashar S., Chik F., Arakelian A., Szyf M., Rabbani S.A. (2015). Pharmacological methyl group donors block skeletal metastasis in vitro and in vivo. Br. J. Pharmacol..

[12] Pakneshan P., Szyf M., Farias-Eisner R., Rabbani S.A. (2004). Reversal of the hypomethylation status of urokinase (uPA) promoter blocks breast cancer growth and metastasis. J. Biol. Chem..

[13] Shukeir N., Pakneshan P., Chen G., Szyf M., Rabbani S.A. (2006). Alteration of the methylation status of tumor-promoting genes decreases prostate cancer cell invasiveness and tumorigenesis in vitro and in vivo. Cancer Res..

[14] Harandi-Zadeh S., Boycott C., Beetch M., Yang T., Martin B.J.E., Ren K., Kwasniak A., Dupuis J.H., Lubecka K., Yada R.Y. (2021). Pterostilbene Changes Epigenetic Marks at Enhancer Regions of Oncogenes in Breast Cancer Cells. Antioxidants.

[15] Beetch M., Boycott C., Harandi-Zadeh S., Yang T., Martin B.J.E., Dixon-McDougall T., Ren K., Gacad A., Dupuis J.H., Ullmer M. (2021). Pterostilbene leads to DNMT3B-mediated DNA methylation and silencing of OCT1-targeted oncogenes in breast cancer cells. J. Nutr. Biochem..

[16] Lubecka K., Kurzava L., Flower K., Buvala H., Zhang H., Teegarden D., Camarillo I., Suderman M., Kuang S., Andrisani O. (2016). Stilbenoids remodel the DNA methylation patterns in breast cancer cells and inhibit oncogenic NOTCH signaling through epigenetic regulation of MAML2 transcriptional activity. Carcinogenesis.

[17] Medina-Aguilar R., Perez-Plasencia C., Marchat L.A., Gariglio P., Garcia Mena J., Rodriguez Cuevas S., Ruiz-Garcia E., Astudillo-de la Vega H., Hernandez Juarez J., Flores-Perez A. (2016). Methylation Landscape of Human Breast Cancer Cells in Response to Dietary Compound Resveratrol. PLoS ONE.

[18] Sigafoos A.N., Paradise B.D., Fernandez-Zapico M.E. (2021). Hedgehog/GLI Signaling Pathway: Transduction, Regulation, and Implications for Disease. Cancers.

[19] Yu F., Yu C., Li F., Zuo Y., Wang Y., Yao L., Wu C., Wang C., Ye L. (2021). Wnt/beta-catenin signaling in cancers and targeted therapies. Signal Transduct. Target. Ther..

[20] Xu X., Zhang M., Xu F., Jiang S. (2020). Wnt signaling in breast cancer: Biological mechanisms, challenges and opportunities. Mol. Cancer.

[21] Pohl S.G., Brook N., Agostino M., Arfuso F., Kumar A.P., Dharmarajan A. (2017). Wnt signaling in triple-negative breast cancer. Oncogenesis.

[22] Riobo-Del Galdo N.A., Lara Montero A., Wertheimer E.V. (2019). Role of Hedgehog Signaling in Breast Cancer: Pathogenesis and Therapeutics. Cells.

[23] Casey S.C., Tong L., Li Y., Do R., Walz S., Fitzgerald K.N., Gouw A.M., Baylot V., Gutgemann I., Eilers M. (2016). MYC regulates the antitumor immune response through CD47 and PD-L1. Science.

[24] Shipitsin M., Campbell L.L., Argani P., Weremowicz S., Bloushtain-Qimron N., Yao J., Nikolskaya T., Serebryiskaya T., Beroukhim R., Hu M. (2007). Molecular definition of breast tumor heterogeneity. Cancer Cell.

[25] Zhang Z.M., Wu J.F., Luo Q.C., Liu Q.F., Wu Q.W., Ye G.D., She H.Q., Li B.A. (2016). Pygo2 activates MDR1 expression and mediates chemoresistance in breast cancer via the Wnt/beta-catenin pathway. Oncogene.

[26] Das S., Samant R.S., Shevde L.A. (2013). Nonclassical activation of Hedgehog signaling enhances multidrug resistance and makes cancer cells refractory to Smoothened-targeting Hedgehog inhibition. J. Biol. Chem..

[27] Ten Haaf A., Bektas N., von Serenyi S., Losen I., Arweiler E.C., Hartmann A., Knuchel R., Dahl E. (2009). Expression of the glioma-associated oncogene homolog (GLI) 1 in human breast cancer is associated with unfavourable overall survival. BMC Cancer.

[28] Peiris-Pages M., Sotgia F., Lisanti M.P. (2015). Chemotherapy induces the cancer-associated fibroblast phenotype, activating paracrine Hedgehog-GLI signalling in breast cancer cells. Oncotarget.

[29] Song L., Li Z.Y., Liu W.P., Zhao M.R. (2015). Crosstalk between Wnt/beta-catenin and Hedgehog/Gli signaling pathways in colon cancer and implications for therapy. Cancer Biol. Ther..

[30] Ding M., Wang X. (2017). Antagonism between Hedgehog and Wnt signaling pathways regulates tumorigenicity. Oncol. Lett..

[31] Xu Y., Yu P., Wang S., Jiang L., Chen F., Chen W. (2019). Crosstalk between Hh and Wnt signaling promotes osteosarcoma progression. Int. J. Clin. Exp. Pathol..

[32] Takada T. (2021). Activation of the Hedgehog and Wnt/beta-Catenin Signaling Pathways in Basal Cell Carcinoma. Case Rep. Dermatol..

[33] Arnold K.M., Pohlig R.T., Sims-Mourtada J. (2017). Co-activation of Hedgehog and Wnt signaling pathways is associated with poor outcomes in triple negative breast cancer. Oncol. Lett..

[34] Farooqi A.A., Khalid S., Ahmad A. (2018). Regulation of Cell Signaling Pathways and miRNAs by Resveratrol in Different Cancers. Int. J. Mol. Sci..

[35] Behroozaghdam M., Dehghani M., Zabolian A., Kamali D., Javanshir S., Hasani Sadi F., Hashemi M., Tabari T., Rashidi M., Mirzaei S. (2022). Resveratrol in breast cancer treatment: From cellular effects to molecular mechanisms of action. Cell. Mol. Life Sci..

[36] Fu Y., Chang H., Peng X., Bai Q., Yi L., Zhou Y., Zhu J., Mi M. (2014). Resveratrol inhibits breast cancer stem-like cells and induces autophagy via suppressing Wnt/beta-catenin signaling pathway. PLoS ONE.

[37] Mo W., Xu X., Xu L., Wang F., Ke A., Wang X., Guo C. (2011). Resveratrol inhibits proliferation and induces apoptosis through the hedgehog signaling pathway in pancreatic cancer cell. Pancreatology.

[38] Gao Q., Yuan Y., Gan H.Z., Peng Q. (2015). Resveratrol inhibits the hedgehog signaling pathway and epithelial-mesenchymal transition and suppresses gastric cancer invasion and metastasis. Oncol. Lett..

[39] Jiang J., Liu Z., Zhou X., Peng F., Wang Z., Li F., Li M. (2022). Resveratrol Induces Apoptosis, Suppresses Migration, and Invasion of Cervical Cancer Cells by Inhibiting the Hedgehog Signaling Pathway. Biomed. Res. Int..

[40] Li W., Cao L., Chen X., Lei J., Ma Q. (2016). Resveratrol inhibits hypoxia-driven ROS-induced invasive and migratory ability of pancreatic cancer cells via suppression of the Hedgehog signaling pathway. Oncol. Rep..

[41] Tost J., Gut I.G. (2007). DNA methylation analysis by pyrosequencing. Nat. Protoc..

[42] Brown S.E., Suderman M.J., Hallett M., Szyf M. (2008). DNA demethylation induced by the methyl-CpG-binding domain protein MBD3. Gene.

[43] Beetch M., Lubecka K., Shen K., Flower K., Harandi-Zadeh S., Suderman M., Flanagan J.M., Stefanska B. (2019). Stilbenoid-Mediated Epigenetic Activation of Semaphorin 3A in Breast Cancer Cells Involves Changes in Dynamic Interactions of DNA with DNMT3A and NF1C Transcription Factor. Mol. Nutr. Food Res..

[44] Cai H., Scott E., Kholghi A., Andreadi C., Rufini A., Karmokar A., Britton R.G., Horner-Glister E., Greaves P., Jawad D. (2015). Cancer chemoprevention: Evidence of a nonlinear dose response for the protective effects of resveratrol in humans and mice. Sci. Transl. Med..

[45] Mai Y., Su J., Yang C., Xia C., Fu L. (2023). The strategies to cure cancer patients by eradicating cancer stem-like cells. Mol. Cancer.

[46] Du J., Johnson L.M., Jacobsen S.E., Patel D.J. (2015). DNA methylation pathways and their crosstalk with histone methylation. Nat. Rev. Mol. Cell Biol..

[47] Vaissiere T., Sawan C., Herceg Z. (2008). Epigenetic interplay between histone modifications and DNA methylation in gene silencing. Mutat. Res..

[48] Tsompana M., Buck M.J. (2014). Chromatin accessibility: A window into the genome. Epigenetics Chromatin.

[49] Cai Y., Zhang Y., Loh Y.P., Tng J.Q., Lim M.C., Cao Z., Raju A., Lieberman Aiden E., Li S., Manikandan L. (2021). H3K27me3-rich genomic regions can function as silencers to repress gene expression via chromatin interactions. Nat. Commun..

[50] Creyghton M.P., Cheng A.W., Welstead G.G., Kooistra T., Carey B.W., Steine E.J., Hanna J., Lodato M.A., Frampton G.M., Sharp P.A. (2010). Histone H3K27ac separates active from poised enhancers and predicts developmental state. Proc. Natl. Acad. Sci. USA.

[51] Zhang T., Zhang Z., Dong Q., Xiong J., Zhu B. (2020). Histone H3K27 acetylation is dispensable for enhancer activity in mouse embryonic stem cells. Genome Biol..

[52] Ramazi S., Zahiri J. (2021). Posttranslational modifications in proteins: Resources, tools and prediction methods. Database.

[53] Ardito F., Giuliani M., Perrone D., Troiano G., Lo Muzio L. (2017). The crucial role of protein phosphorylation in cell signaling and its use as targeted therapy (Review). Int. J. Mol. Med..

[54] Shah K., Kazi J.U. (2022). Phosphorylation-Dependent Regulation of WNT/Beta-Catenin Signaling. Front. Oncol..

[55] Gao C., Xiao G., Hu J. (2014). Regulation of Wnt/beta-catenin signaling by posttranslational modifications. Cell Biosci..

[56] Qadir Nanakali N.M., Maleki Dana P., Sadoughi F., Asemi Z., Sharifi M., Asemi R., Yousefi B. (2023). The role of dietary polyphenols in alternating DNA methylation in cancer. Crit. Rev. Food Sci. Nutr..

[57] Hoffmann A., Meir A.Y., Hagemann T., Czechowski P., Muller L., Engelmann B., Haange S.B., Rolle-Kampczyk U., Tsaban G., Zelicha H. (2023). A polyphenol-rich green Mediterranean diet enhances epigenetic regulatory potential: The DIRECT PLUS randomized controlled trial. Metabolism.

[58] Arora I., Sharma M., Tollefsbol T.O. (2019). Combinatorial Epigenetics Impact of Polyphenols and Phytochemicals in Cancer Prevention and Therapy. Int. J. Mol. Sci..

[59] Zhang Q., Pan Y., Ji J., Xu Y., Zhang Q., Qin L. (2021). Roles and action mechanisms of WNT4 in cell differentiation and human diseases: A review. Cell Death Discov..

[60] Vouyovitch C.M., Perry J.K., Liu D.X., Bezin L., Vilain E., Diaz J.J., Lobie P.E., Mertani H.C. (2016). WNT4 mediates the autocrine effects of growth hormone in mammary carcinoma cells. Endocr. Relat. Cancer.

[61] Kreibich E., Kleinendorst R., Barzaghi G., Kaspar S., Krebs A.R. (2023). Single-molecule footprinting identifies context-dependent regulation of enhancers by DNA methylation. Mol. Cell.

[62] Li Y., Chen X., Lu C. (2021). The interplay between DNA and histone methylation: Molecular mechanisms and disease implications. EMBO Rep..

[63] Wang X., Xu J., Sun Y., Cao S., Zeng H., Jin N., Shou M., Tang S., Chen Y., Huang M. (2023). Hedgehog pathway orchestrates the interplay of histone modifications and tailors combination epigenetic therapies in breast cancer. Acta Pharm. Sin. B.

[64] Sharma A., Mir R., Galande S. (2021). Epigenetic Regulation of the Wnt/beta-Catenin Signaling Pathway in Cancer. Front. Genet..

[65] Wils L.J., Bijlsma M.F. (2018). Epigenetic regulation of the Hedgehog and Wnt pathways in cancer. Crit. Rev. Oncol. Hematol..

[66] Yang D., Li Q., Shang R., Yao L., Wu L., Zhang M., Zhang L., Xu M., Lu Z., Zhou J. (2020). WNT4 secreted by tumor tissues promotes tumor progression in colorectal cancer by activation of the Wnt/beta-catenin signalling pathway. J. Exp. Clin. Cancer Res..

[67] Ma J., Huang X., Li Z., Shen Y., Lai J., Su Q., Zhao J., Xu J. (2019). FOXE1 supports the tumor promotion of Gli2 on papillary thyroid carcinoma by the Wnt/beta-catenin pathway. J. Cell. Physiol..

[68] Peng Y., Zhang X., Lin H., Deng S., Qin Y., He J., Hu F., Zhu X., Feng X., Wang J. (2021). Dual activation of Hedgehog and Wnt/beta-catenin signaling pathway caused by downregulation of SUFU targeted by miRNA-150 in human gastric cancer. Aging.

[69] Bracci L., Fabbri A., Del Corno M., Conti L. (2021). Dietary Polyphenols: Promising Adjuvants for Colorectal Cancer Therapies. Cancers.

[70] Jakobusic Brala C., Karkovic Markovic A., Kugic A., Toric J., Barbaric M. (2023). Combination Chemotherapy with Selected Polyphenols in Preclinical and Clinical Studies-An Update Overview. Molecules.

[71] Ahmad J., Ahamad J., Algahtani M.S., Garg A., Shahzad N., Ahmad M.Z., Imam S.S. (2024). Nanotechnology-mediated delivery of resveratrol as promising strategy to improve therapeutic efficacy in triple negative breast cancer (TNBC): Progress and promises. Expert Opin. Drug Deliv..

[72] Wu X.L., Lin S.G., Mao Y.W., Wu J.X., Hu C.D., Lv R., Zeng H.D., Zhang M.H., Lin L.Z., Ouyang S.S. (2023). Wnt/beta-catenin signalling pathway in breast cancer cells and its effect on reversing tumour drug resistance by alkaloids extracted from traditional Chinese medicine. Expert Rev. Mol. Med..

[73] Li F., Han Y., Wu X., Cao X., Gao Z., Sun Y., Wang M., Xiao H. (2022). Gut Microbiota-Derived Resveratrol Metabolites, Dihydroresveratrol and Lunularin, Significantly Contribute to the Biological Activities of Resveratrol. Front. Nutr..

[74] Springer M., Moco S. (2019). Resveratrol and Its Human Metabolites-Effects on Metabolic Health and Obesity. Nutrients.

[75] Guthrie A.R., Chow H.S., Martinez J.A. (2017). Effects of resveratrol on drug- and carcinogen-metabolizing enzymes, implications for cancer prevention. Pharmacol. Res. Perspect..

