# Supplementary material for: Epigenetic Effects of Resveratrol on Oncogenic Signaling in Breast Cancer
Source: Nutrients. 2024 Feb 29;16(5):699. doi: 10.3390/nu16050699 (PMC10934115; doi:10.3390/nu16050699)
Supplement: Supplementary file 1 [file nutrients-16-00699-s001.zip › nutrients-2873629-supplementary.pdf]

“Epigenetic effects of resveratrol on oncogenic signaling in breast cancer”
Lucinda Kurzava Kendall

Supplementary Data

Supplementary Figure S1: Resveratrol (RSV) exerts a dose-dependent effect on growth of breast cancer cells. Resveratrol (RSV) exerts a dose-dependent effect on growth of breast cancer cells. Effect on cell growth after 4- and 9-day treatments with RSV at 1–25 µM concentrations in MCF10CA1a (data adapted from Ref 16) and MCF-7 breast cancer cells, as measured with a trypan blue exclusion test. Cell number is expressed as a percentage of control (cells treated with vehicle-ethanol). IC50, which refers to a non-cytotoxic dose causing a 50% decrease in cell growth and less than 10% cell mortality, was determined to be between 21-22µM at day 4 and 15µM at day 9 in both cell lines. All results represent mean ± SD of three independent experiments.

"Epigenetic effects of resveratrol on oncogenic signaling in breast cancer"
Lucinda Kurzava Kendall

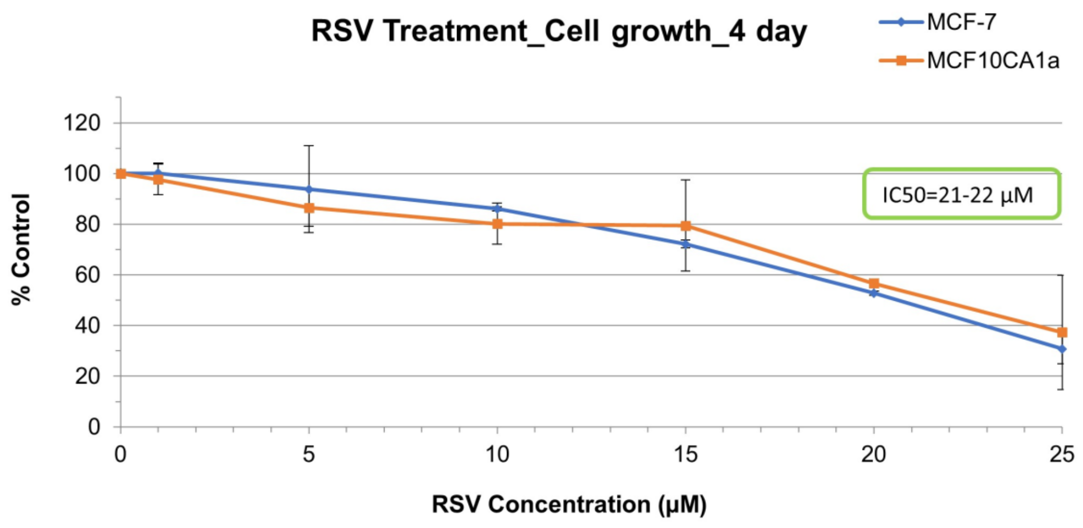

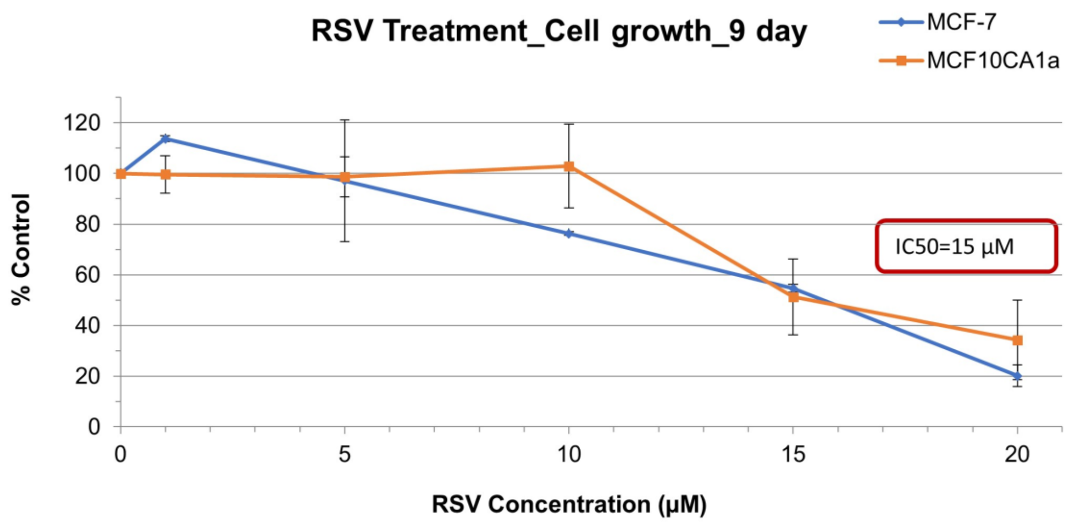

Supplementary Table S1. Pyrosequencing, qRT-PCR and quantitative ChIP (qChIP) primer sequences for the selected gene targets.

Supplementary Table S1. Primer sequences used in qRT-PCR, pyrosequencing and qChIP.

Gene	Primer sequences	Annealing temperature [°C]
**qRT-PCR**		
GLI2	FW 5'-GGTACCATTACGAGCCTCATTC-3'<br>RV 5'-AAAGCCGGATCAAGGAGATG-3'	59
WNT4	FW 5'-CCTTCGTGTACGCCATCTCT-3'<br>RV 5'-TCAGAGCATCCTGACCACTG-3'	59
BCL2	FW 5'-CAGAAGTCTGGGAATCGATCTG-3'<br>RV 5'-AATCTTCAGCACTCTCCAGTTATAG-3'	59
EpCAM	FW 5'-CCAGAACAATGATGGGCTTTATG-3'<br>RV 5'-CAGTGTCCTTGTCTGTTCTTCT-3'	59
CCND1	FW 5'-AAATGTGTGCAGAAGGAGGTC-3'<br>RV 5'-AGCGGTCCAGGTAGTTCAT-3'	59
CYR61	FW 5'-GGCAGACCCTGTGAATATAACT-3'<br>RV 5'-ACGGCGCCATCAATACAT-3'	59
**Pyrosequencing**		
GLI2	FW 5'-AGGAGGTATTGGGGAAGTTAT-3'<br>RVBio 5'-CATTCCCCTAATCATCTATAAATTACAATC-3'<br>Seq 5'-GTATTGGGGAAGTTATAGT-3'	53.5
WNT4	FW 5'- GAAGGGATTTGGTTTTAGTTATTTAGT-3'<br>RVBio 5'- CCAACCCACCATACATACTCCAACTCA-3'<br>Seq1 5'- ATGTTTTTATTTTTTTAAGAATTGT-3’<br>Seq2 5'- AGAGAGTTATGGAATTAAGATTTA-3’	49
**qChIP**		
GLI2	FW 5'-TTCTAGCATGCAAGCCTGTG-3'<br>RV 5'-GGACTTGCTGGAGCACAC-3'<br><br>FW 5'-CGTGTGCTCCAGCAAGT-3'<br>RV 5'-TAGTCAGTGAATGCAGAGTGAAA-3'<br><br>FW 5'-TGCAGCTGCTTCACCATAAA-3'<br>RV 5'-ATGTGACCTCAGGCCCTTAT-3'<br><br>FW 5'-CTGAGAAGGGTGGTTCTGTTAG-3'<br>RV 5'-GGTCAGGATTCAAGGCAAGT-3'	59

	FW 5'-CCCTCTGTGTCCTCCTTCTTA-3'<br>RV 5'-AAAGCCGGATCAAGGAGATG-3'<br><br>FW 5'-CACGCTCTCCATGATCTCTG-3'<br>RV 5'-GCTGAGGAGTTGACAGTGTTAG-3'	
WNT4	FW 5'-CCTCGCCTGAATGGATGTT-3'<br>RV 5'-CTCCAGAAAGAGAGTCCACAAC-3'<br><br>FW 5'-TGGCTTCAGAGCTTGTGTT-3'<br>RV 5'-GCCTGTCTGGTCTTCCATAAA-3'<br><br>FW 5'-GAGGAGTTGCCCTTCCTTATG-3'<br>RV 5'-GGGTTACGTAGAAGCTGTTGT-3'<br><br>FW 5'-TGGTTGGCAATTACCTCTGT-3'<br>RV 5'-GCACAGAGAAGGTGATTGTTTC-3'<br><br>FW 5'-AGACAGAGTCTTCTCCGAGTTA-3'<br>RV 5'-TGGTTCAGGCCTGGAAATAC-3'	59
